# FUSION regimen: ranibizumab in treatment-naïve patients with exudative age-related macular degeneration and relatively good baseline visual acuity

**DOI:** 10.1007/s00417-012-2009-5

**Published:** 2012-04-15

**Authors:** Jordi Monés, Marc Biarnés, Fabio Trindade, Ricardo Casaroli-Marano

**Affiliations:** 1 Institut de la Màcula i de la Retina, Centro Médico Teknon, Vilana 12, Barcelona, 08022 Spain; 2 Departamento de Cirugía, Facultat de Medicina, Universitat de Barcelona, Barcelona, Spain

**Keywords:** Exudative age-related macular degeneration, Ranibizumab, PRN regimen, Anti-VEGF therapy, FUSION, Treat-and-extend regimen

## Abstract

**Background:**

To investigate the safety and efficacy of a combined fixed-interval and *pro re nata* regimen of ranibizumab (FUSION regimen) for treatment of exudative age-related macular degeneration in patients with good visual acuity at baseline. To establish whether similar efficacy to monthly regimens can be achieved with fewer injections, even in patients with good visual acuity.

**Methods:**

This was a prospective, open-label, consecutive interventional case series in treatment-naïve patients with exudative age-related macular degeneration. The FUSION regimen consists of three phases: 1) a loading phase of two or three injections, depending on presence or absence of choroidal neovascularization activity at first follow-up, 2) administration of one injection on disappearance of exudation, and 3) subsequent administration of two separate injections at intervals 2 months apart, and then an injection every 3 months. Endpoints included visual acuity, presence of fluid, adverse events and number of injections administered.

**Results:**

Seventeen eyes of 17 Caucasian patients were included. Mean patient age was 76 years, and 15 patients were female. Mean baseline visual acuity was 67.5 letters (median 67), with Snellen equivalent 20/50++, ranged between 45 (20/125) and 83 (20/20−−). At 3 months, mean change in best-corrected visual acuity (BCVA) was +2.3 letters (median +9) compared with baseline (*p* = 0.3). At 6 months, mean change in BCVA was +4.2 letters (median +9) compared with baseline (*p* = 0.02). At 12 months, one patient had discontinued the study. Mean change in BCVA was 5.6 (median +10) compared with baseline (*p* = 0.04). No patient lost ≥15 letters, and 14 patients (87.5%) lost <5 letters. The mean number of injections was 6.9. One patient experienced a retinal pigment epithelium tear; no other complications were observed.

**Conclusions:**

The FUSION regimen for ranibizumab has the potential to maintain visual gains achieved during the loading phase, as reported in studies with monthly injections, even in eyes with a relatively good visual acuity at baseline. These 12-month results warrant validation in a larger, randomized controlled trial.

## Introduction

The approval of ranibizumab (Novartis Pharma AG) marked a significant change in treatment outcomes for exudative age-related macular degeneration (AMD), and established a new standard of care for the treatment of this disabling disease. Although monthly injections of ranibizumab resulted in the highest efficacy [1], concerns were raised regarding potential ocular and systemic safety risks, and feasibility and costs.

**Fig. 1:**
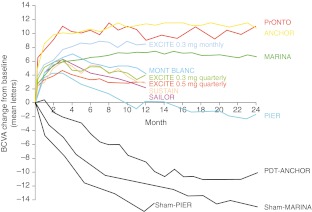
Summary of mean BCVA change from baseline over time in patients treated with ranibizumab. Clinical trial results shown were achieved with various ranibizumab treatment regimens [17]. (Reproduced with permission from *Ophthalmologica*)

Alternative regimens attempting to achieve similar efficacy with fewer injections of ranibizumab have been explored. In the PIER [2] and EXCITE studies [3, 4] mean best-corrected visual acuity (BCVA) improved after three initial monthly injections; however, this declined during the subsequent fixed-quarterly injection phase (Fig. 1).

Trials investigating *pro re nata* (PRN) regimens [5–9] also showed loss of mean BCVA from the peak value reached after monthly initiation phase. Furthermore, PRN regimens all rely on monthly follow-up, which is not usually achieved in the ‘real-world’ setting. Furthermore, PRN regimens with retreatment only administered when lesion activity recurs (i.e., a visual acuity [VA] loss of ≥5 letters) might be associated with a risk of irreversible visual loss that is significant [10].

In the recently reported CATT study, PRN ranibizumab barely achieved non-inferiority to monthly treatment with 24.9 % vs 34.2 % of these groups respectively, gaining >15 letters (*p* = 0.09) [11]. PRN ranibizumab led to less reduction of retinal thickness and a numerically lower functional gain compared with monthly treatments. Moreover, at week 36, the plotted mean changes in VA for the PRN arms start to diverge downhill in comparison with the monthly arms, suggesting that, after 12 months, the differences in benefit might increase.

Regimens such as ‘inject and extend’ or ‘treat and extend’, in which the intervals between re-injections are progressively extended if there is no fluid, appear to be more favourable than with PRN regimens [12–16]. However, these regimens carry the risk of long-term unnoticed disease recurrence, and require understanding of the algorithm by both patient and practitioner to manage frequent changes to the visit schedule.

The rationale for the ‘FUSION’ regimen that combines fixed injections after defined periods of (apparent) inactivity with the PRN approach has been reported [17]. The FUSION regimen will be practical enough to be broadly implemented into clinical routine.

Here we present a small proof-of-concept study, designed to explore the efficacy of the FUSION regimen. The main objective is to ascertain if this FUSION regimen will be effective enough in the current incident population presenting in clinical practice who have higher baseline BCVA than patients in most previous trials.

## Materials and methods

This was a prospective, open-label, consecutive interventional case series of a 12-month study of patients with exudative AMD treated with intravitreal injections of 0.5 mg ranibizumab in a combined fixed-interval and PRN regimen. The 12-month results reported in this work include only patients enrolled during November and December 2010. Ranibizumab (LUCENTIS®; Novartis Farmacéutica SA, Spain) is approved by the European Medicines Agency and the US Food and Drug Administration, and is the current standard of care for treatment of exudative AMD.

The study adhered to the tenets of the Declaration of Helsinki. The protocol was approved by an Ethics Committee, and informed consent was obtained from all patients. All patients were given an explanation of the trial, and information about possible risks and discomforts by the investigator. Patients who provided written informed consent and met all eligibility criteria were enrolled into the study. All participants were scheduled to be followed up monthly; in cases where the choroidal neovascularization (CNV) had been inactive for 6 months, follow-up visits were held at 1.5-month intervals.

## General inclusion and exclusion criteria

Patients included in the study were of either gender, were aged ≥50 years, provided written informed consent, and were capable of complying with all study and follow-up procedures and of returning for all visits.

Patients were excluded from the study if they: had a history or evidence of severe cardiac disease; had experienced a stroke within the 6 months preceding trial entry; had undergone any major surgical procedure within 1 month of study entry or any treatment with an investigational agent in the 60 days prior to the beginning of the study; or had known serious allergies to the fluorescein dye used in angiography (mild allergy amenable to treatment was permitted), or to the components of the ranibizumab formulation.

## Ophthalmic inclusion and exclusion criteria

The following inclusion criteria were applied to the study eye: (1) subfoveal or juxtafoveal CNV owing to AMD, defined by fluorescein angiography (FA), (2) presence on optical coherence tomography (OCT) of subretinal or intraretinal fluid associated or not with macular oedema, (3) BCVA in the study eye between 20/20 and 20/125, inclusive, (4) total area of the lesion (including blood, neovascularization and scar/atrophy) of ≤8 disc areas, of which at least 50% must be active CNV (defined as the neovascular component of the lesion as defined by FA; all angiographic subtypes [predominantly classic, minimally classic and occult] were eligible), (5) clear ocular media and adequate pupillary dilatation to allow collection of fundus photographs and FA of a sufficient quality to be analysed; intraocular pressure of 21 mmHg or less, and (6) no previous treatment for AMD.

Ophthalmic exclusion criteria comprised: (1) presence of scarring or atrophy >75% of the total lesion size (patients with subfoveal scar or atrophy were excluded), (2) subretinal haemorrhage >75% of the total lesion size, (3) presence of serous retinal pigment epithelial detachments >5 disc areas, (4) presence of intraocular inflammation (≥ trace cell or flare), epiretinal membrane, macular hole or vitreous haemorrhage, (5) history of idiopathic or autoimmune-associated uveitis in either eye, (6) significant media opacities, including cataract, which might interfere with VA, assessment of toxicity, or fundus photography in the study eye, (7) presence of other causes of CNV, including pathological myopia (spherical equivalent of −3 diopters or more, or axial length of 25 mm or more, or fundus findings suggestive of pathologic myopia), ocular histoplasmosis syndrome, angioid streaks, choroidal rupture and multifocal choroiditis, (8) any retinal treatment (aside from antioxidants), including (but not limited to) intravitreal injections, photodynamic therapy with verteporfin, laser photocoagulation, or surgery, (9) history of rhegmatogenous retinal detachment, pars plana vitrectomy or corneal transplant, and (10) previous radiation in the region of the study eye.

## Study protocol and assessments

Consenting patients were screened for the study with a medical and ophthalmological history, measurement of BCVA, slit-lamp examination, measurement of intraocular pressure, and funduscopic examination with pharmacological pupil mydriasis (one drop phenylephrine 10% plus tropicamide 1%). In addition, colour fundus photographs were obtained with non-stereoscopic 35° photography of field 2 (Topcon TRC 50DX IA®, Topcon Corporation, Tokyo, Japan), and FA (Spectralis HRA + OCT®, Heidelberg Engineering, Heidelberg, Germany) and spectral-domain OCT (SD OCT; Spectralis HRA + OCT®, Heidelberg Engineering) were carried out. Safety evaluations, measurement of BCVA, eye examinations, and OCT scans were conducted at all study visits. FA was performed at least at baseline, months 1 and 3, and at those visits where injections were not predetermined.

## Regimen (FUSION) and administration of intravitreal drug

The FUSION regimen consists of three steps. First, a loading phase of three consecutive monthly injections is given. If CNV activity is resolved at the first follow-up visit, the loading phase can be reduced to two monthly injections. Second, a PRN regimen is established on demand, and intravitreal injections are given if CNV activity is present, defined by fluorescein leakage at FA and/or the presence of abnormal intraretinal or subretinal fluid assessed by OCT, until cessation of activity. After cessation of CNV activity, patients still receive one injection. Third, after cessation of CNV activity, patients receive fixed injections every 2 months for two courses, and every 3 months for two courses.

**Fig. 2:**
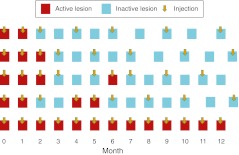
The FUSION regimen: patients are treated by a combination of PRN and fixed-dose injections according to lesion activity. Five examples of the FUSION regimen are represented in the figure [17]. (Reproduced with permission from *Ophthalmologica*)

At the intermediate visits, between the preplanned fixed injections, patients are treated according to PRN criteria. The PRN intermediate visits are held at 4-weekly intervals during the first two periods of fixed injections every 2 months, and at 6-weekly intervals when fixed injections are given every 3 months. Treatment is restarted at the first sign of CNV lesion activity (as defined in the Ophthalmic inclusion and exclusion criteria section) at the intermediate visits. The retreatment cycle is restarted in cases of significance relapse during the period of fixed injections, until cessation of CNV activity. If a patient never shows resolution of fluid, injections according to PRN criteria would be given at every visit (Fig. 2).

Intravitreal therapy was carried out in sterile conditions after topical anaesthesia, and the injection site was cleaned with 5 % povidone iodine. Lid speculum was located, and additional topical anaesthesia and povidone iodine were administered immediately prior to injection. Ranibizumab (0.5 mg/0.05 ml) was injected through the pars plana at 4 mm of limbus into the vitreous cavity. Patients were instructed to consult the study team if they experienced decreased vision, eye pain, unusual redness or any new symptoms.

## Safety and efficacy endpoints

All adverse events were registered. Ophthalmic variables, such as intraocular pressure, general ophthalmic examination, colour fundus photography, and OCT, were observed at each follow-up visit. FA was carried out at least at month 3 and at those visits where fixed injections were not predetermined.

The primary outcome measure was the change in BCVA between baseline and months 3, 6, and 12. Secondary vision-related outcome measures were the percentage of patients with gain of ≥5, >10, and ≥15 letters ETDRS at 12 months. The percentage of patients losing <5, <15, and <30 letters at 12 months was also observed. In addition, the following endpoints were also considered: the mean and median VA at 6 and 12 months, and the mean number of injections at 12 months.

## Statistical analysis

Descriptive statistics included age, gender and baseline BCVA. Median BCVA change was assessed using the Wilcoxon test. The total number of intravitreal injections was also considered, as was the frequency with which treatment was required at the PRN visits that was not anticipated by the FUSION schedule at the predetermined fixed treatment visits. Owing to the high percentage of patients with VA >20/40, statistical analysis was also carried out by subgroup of percentages of patients who maintained VA, lost ≥3 lines of VA or gained ≥3 lines of VA, including the group of patients with basal BCVA ≥20/40 and the group of patients with basal BCVA <20/40.

All adverse events occurring up to 12 months after the first injection were included. Stata IC (version 11.1; Stata Corp, College Station, TX, USA) was used for statistical analysis. A *p*­value <0.05 was considered statistically significant.

## Results

The study included 17 eyes of 17 treatment-naïve Caucasian patients (15 female) with a mean age of 76 years (range 61 to 87 years). The right eye was treated in four cases (23.5 %). Mean baseline BCVA was 67.5 letters (median 67, Snellen equivalent 20/50++, range 45 letters [20/125] to 83 letters [20/20−−]). Approximately 50% of the patients had a VA >73 letters (20/40 Snellen) with a mean VA of 82 letters (20/25++).

At 3 months, the mean change in VA was +2.3 letters (*p* = 0.3) from baseline (median +9 letters). At 6 months, the mean change in VA was +4.2 letters (*p* = 0.02) from baseline (median +9 letters). At 12 months, the mean change in VA was +5.6 letters (*p* = 0.04) from baseline (median +10 letters).

Mean BCVA at 6 and 12 months were 71.7 and 73.1 letters (corresponding to 20/40++ and 20/32−− respectively). Median BCVA at 6 and 12 months were 76.5 and 77.5 letters (corresponding to 20/32++ and 20/25−− respectively).

All 17 patients were followed-up until 6 months. At 12 months, one patient had discontinued the study. Out of a total of 170 permissible visits, 155 were performed (91.2 %). No patient lost ≥15 letters, and vision was maintained in 14 patients (87.5 %), defined as loss <5 letters. Vision was maintained in seven out of eight patients (87.5 %) with VA at baseline <20/40, and in seven out of eight patients (87.5 %) with baseline VA ≥20/40. At 12 months, nine of the 16 patients (56.3 %) gained ≥5 letters: seven of the eight patients (87.5 %) with baseline VA <20/40, and two of the eight patients (25.0 %) with a baseline VA ≥20/40. Two of the 16 patients (12.5 %) experienced an improvement in VA of ≥15 letters: two out of the eight patients (25 %) with a baseline VA <20/40. Six of the 16 patients gained >10 letters (37.5 %): six of the eight patients (75.0 %) with baseline VA <20/40.

The mean number of injections at month 12 was 6.9 out of 12 possible (median 7, ranging from 5 to 10). Eleven patients (64.7%) required the three loading injections; six patients had inactive CNV (resolution of fluid) after one injection and thus received only one more injection in the loading phase, i.e., the third injection was postponed by 1 month, initiating the fixed bimonthly injections. PRN retreatment outside the fixed schedule had to be performed in three of the 16 patients (18.8 %). In 29 of the 69 (42.0 %) who were administered fixed injections, the lesion did not present active exudation at OCT and/or FA.

One patient (5.9 %) in the overall study population experienced a retinal pigment epithelium tear after the first intravitreal injection. No other adverse events were observed.

## Discussion

The objective of this pilot study was to explore the safety and efficacy of the FUSION regimen, which presents an alternative to monthly injections, in a population of patients with a high BCVA score, and therefore at a high risk of vision loss. This high-baseline BCVA is consistent with the BCVA of patients at initial presentation in clinical practice, a scenario that has not been addressed in previous trials. If this regimen could show benefit even in this particular population, further research with larger clinical trials would be recommended.

At 6 months, an interim analysis showed no safety signals that would prohibit continuation of the study. In terms of efficacy, the following trend was discovered: BCVA gains after the loading dose could be maintained from 1 month after the loading dose to month 6. This trend may suggest superiority compared with regimens that apply PRN criteria (i.e., retreatment is triggered by recurrent disease activity), because most reports describing PRN regimens show BCVA loss from the values obtained after the loading dose. Therefore, the study was continued to month 12.

**Fig. 3:**
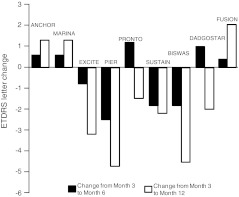
Change in visual acuity from month 3 to month 6 and from month 3 to month 12 in the different trials. Only MARINA, ANCHOR, PrONTO, Dadgostar and FUSION showed a positive change from month 3 to month 6. Only MARINA, ANCHOR and FUSION showed a positive change from month 3 to month 12, despite the mean visual acuity at baseline in FUSION being 10–20 letters higher than in these studies

**Fig. 4:**
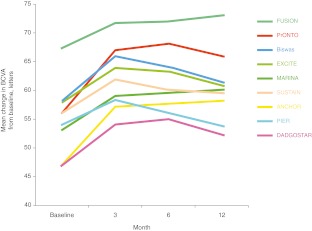
Mean change from baseline in visual acuity observed in the different studies. Despite the high level of visual acuity at baseline in the FUSION series, mean visual acuity gain is obtained at 3, 6, and 12 months

Mean change in VA at 12 months using the FUSION regimen was a gain of 5.6 letters, despite the relatively good baseline VA, and is within the range of VA gain shown by other trials including patients with a higher mean VA at baseline. After administration of ranibizumab according to the FUSION regimen, no patient experienced severe or moderate visual loss (loss of ≥30 or ≥15 letters, respectively). Fourteen patients (87.5 %) did not experience any significant loss of vision, defined as loss of <5 letters. It is noteworthy that this level of maintaining vision was also achieved for the subgroup of patients with VA ≥20/40. In addition, 56.3 % of patients had gain of vision of ≥5 letters; 25.0 % of those patients with VA of ≥20/40 also had gain of ≥5 letters. Owing to the good level of VA at baseline, it was anticipated that significant VA gain of three lines or more would be less frequent than in other trials with much lower VA at baseline. Two of the 16 patients (12.5 %) gained three lines of VA. Both of these patients had VA <20/40 at baseline, representing 25.0 % of this subgroup. Six of the 16 patients (37.5%) gained >10 letters: 75.0 % of those patients with VA <20/40. The proportion of patients with ≥15 letter gain (12.5 %) may appear smaller in comparison to those in the MARINA [18] and ANCHOR [19] studies. This could be explained by the fact that the patients described here had a baseline BCVA of 67.5 letters, and half of the patients had a VA >73 letters (20/40 Snellen) with a mean VA of 82 letters (20/25++). This baseline VA is in the order of 10–20 letters higher than the baseline VA in other trials (Table 1, Figs. 3 and 4). Patients with high baseline VA are less likely to gain BCVA and are at risk of higher BCVA loss [20, 21].

**Table 1:** Main characteristics of different trials and clinical series. Mean baseline visual acuity for the FUSION regimen was 10–20 letters higher than in other studies. Despite this higher level of baseline mean visual acuity, patients showed a mean gain of 2.3 letters at month 3, 4.2 letters at month 6, and 5.6 letters at month 12. There was no loss between months 3 and 6, and month 3 and month 12

Trial	Patients treated (*n*)	Regimen	Baseline VA	Δ3M VA	Δ6M VA	Δ12M VA	Diff. Δ6–Δ3M	Diff. Δ12–Δ3M	Mean injections (*n*)
ANCHOR [19]	140	Monthly	47	10.0	10.6	11.3	0.6	1.3	12.0
MARINA [18]	240	Monthly	53	5.9	6.5	7.2	0.6	1.3	12.0
EXCITE [4]	118	3 + quarterly	58	6.0	5.2	2.8	−0.8	−3.2	5.5
PIER [2]	61	3 + quarterly	54	4.5	2.0	−0.2	−2.5	−4.7	6.0
PrONTO [5]	40	3 + PRN (5 letter / 100 μm / fluid)	56	10.8	12.0	9.3	1.2	−1.5	5.6
SUSTAIN [23]	513	3 + PRN (5 letter / 100 μm / fluid)	56	5.8	4.0	3.6	−1.8	−2.2	5.6
Biswas [24]	27	3 + PRN (5 letter / 100 μm)	58	5.8	5.7	3.2	−0.1	−2.6	5.6
Dadgostar [8]	124	1 + PRN (OCT, clinical)	47	7.0	8.0	5.0	1.0	−2.0	5.2
FUSION	17	2–3 + fixed bi- or 3­monthly + PRN (OCT, FA)	67	2.3	4.2	5.6	1.9	3.3	6.9

Δ3M VA, change in mean visual acuity at 3 months; Δ6M VA, change in visual acuity at 6 months; Δ12M VA, change in visual acuity at 12 months; Diff. Δ6–Δ3M, difference in mean visual acuity from month 3 to month 6; Diff. Δ12–Δ3M, difference in visual acuity from month 3 to month 12; FA, fluorescein angiography; OCT, optical coherence tomography; PRN, *pro re nata*; VA, visual acuity

The mean number of injections (6.9 out of 12 possible) is within the value that could be expected for PRN regimens. The pre-planned schedule of fixed injections was appropriate for the majority of patients, with few patients requiring additional injections. However, 18.8 % of patients who had resolution of fluid required injection at those visits where treatment had not been predetermined by a fixed administration. These patients would have been missed by the ‘treat and extend’ regimens, where these intermediate visits between injections do not take place. Furthermore, the timings of the fixed injections do not appear to be exaggerated, because approximately half of the patients presented with some exudation at those visits.

Perhaps an indication of the success of the FUSION regimen in the population of patients with a higher risk of losing vision is the fact that 42 % of the fixed injections were performed in inactive lesions. This might suggest that the regimen anticipates the relapse of neovascular exudation.

These data indicate that the FUSION regimen, with its fixed injections, sufficiently stabilizes the disease in most patients, but has the flexibility to counteract acute and unexpected disease recurrence via the PRN injections (Fig. 2). Therefore, the FUSION regimen is flexible enough to spare injections in patients who have a more favourable response to treatment and also allows, at the other extreme of the spectrum, monthly treatments if needed (Fig. 2). Compared with PRN regimens, treatment before the recurrence of CNV activity appears to lower the risk of losing vision or losing the vision that is gained initially.

Several FA evaluations were performed during the FUSION study; however, the availability of SD OCT has greatly reduced the number of angiographies performed in clinical practice and could potentially mean that, in the future, angiography as an invasive technique will be no longer considered as necessary in the follow-up of patients with exudative AMD. At present, there is no consensus on this matter; thus, the use of FA in addition to SD OCT is based on clinical judgement of the retinal specialists, and varies between centres and countries. Although this question was not specifically addressed in the FUSION study, in our practice, FA is very useful in providing an entire picture of the lesion, particularly in cases of questionable OCT findings in chronic lesions, or specially in juxtafoveal lesions where recurrences may be questionable at OCT but very evident in FA.

The duration of this study was only 12 months, whereas PrONTO [5], ANCHOR [19] and MARINA [18] showed that there was a benefit in treating for 2 years. Thus, the FUSION regimen, if validated, should be followed for at least 2 years. The HORIZON trial [22] (using a PRN regimen) has shown that continued treatment is needed in a large proportion of patients in the third and fourth year, and that less frequent dosing in years 3 and 4 was associated with visual decline. Therefore, a PRN regimen is likely to be insufficient even after 2 years.

Although conceptually different, the FUSION regimen has some similarities with the proposed ‘treat and extend’ regimen [12–16], because both regimens aim to prevent disease recurrence. The main differences between the FUSION and ‘treat and extend’ regimens are that, in the latter, the periods between treatments are extended in a continuous linear form, and there are no visits in between these periods. Long extension, however, bears an increased risk of unnoticed disease reactivation. In at least 18 % of the patients in this study, the ‘treat and extend’ regimen would have missed early detection of relapse of exudation. The goal of the FUSION regimen is to treat before relapse of neovascular activity, ideally in an inactive lesion.

The lack of a control group and the small sample size are the main limitations of this study; however, these are limitations inherent in pilot studies.

The purpose of this exploratory 12-month study was not to establish a recommendation for using the FUSION regimen, but to provide sufficient evidence to justify and raise a proposal for a larger, randomized controlled trial. This trial would aim to establish the exact role of the FUSION approach, and to validate if it can be used as an alternative to monthly regimens without compromising the extent of vision gain, even in the current population of patients in daily clinical practice who may present with very good levels of vision.
